# Section on Obstetrics and Diseases of Women

**Published:** 1881-06

**Authors:** 


### FIRST DAY.

SECTION ON OBSTETRICS AND DISEASES OF WOMEN.—Chairman, Dr. James R. Chadwick, of Boston; Secretary, Dr. Joseph Tabor Johnson, D. C.

*Résumé of Rules for the use of Pessaries.*—Dr. Paul F. Mundé, of New York, made a brief recapitulation of the rules governing the introduction and supervision of vaginal pessaries (including vagino-abdominal).

1. Always be sure of the diagnosis of the nature and degree of the displacement before resorting to a pessary.

2. Always replace the uterus before applying a pessary. This applies particularly to retro-displacements. It is well to replace the uterus repeatedly, every day or twice daily, for several days before introducing a pessary. The replaced organ may be supported by a cotton tampon in the interval, if it is desired to distend and toughen the vaginal pouch; or the object of relaxing the abnormally stretched uterine ligaments may have been obtained by the mere repeated replacement. In flexions, chiefly ante-flexions, the frequent straightening of the uterus, or conversion into the opposite flexion by the sound, will often prove beneficial before introducing a pessary.

3. Never insert a pessary when there is evidence, by the touch, of acute or recent inflammation of the uterus or adnexa, or when pressure by the finger on the parametrium (where the pessary is to rest) gives decided pain.

4. When the uterus is not replaceable—that is, when adhesions bind the fundus down—use great caution and discrimination in deciding whether an attempt should be made and is justified by the symptoms, to elevate the fundus by manual or instrumental means, or whether the elevation should first be tried by the gradual elevation of a pessary (this applies only to retro- and latero-versions). If neither is to be recommended, do not introduce a pessary until local alterative and absorbent measures have affected a resolution of the adhesions.

5. Always choose an indestructible instrument, if possible. This does not apply to prolapsus uteri.

6. Always measure and estimate the vagina carefully before choosing a pessary, and be careful to adjust the pessary in every particular (size, curve, width) to that particular case. No two vaginæ are exactly alike.

7. If the vaginal pouch is not sufficiently deep to accommodate a pessary (anterior pouch for ante-displacements; posterior pouch for retro-displacements), defer the attempt to fit a pessary until the pouch has been deepened by daily tamponing with cotton, or by the upward pressure of a Cutter or Thomas vagino-abdominal supporter. Or the pouch may be gradually deepened by using first a small (slightly curved in retro-displacement) instrument, and gradually increasing its size for curve, until the desired size and shape for permanency is reached.

8. Never leave a pessary in the vagina which puts the vaginal walls to the stretch, and which does not permit the passage of a finger between it and the wall of the vagina. This does not apply to prolapsus uteri.

9. A vaginal pessary which projects from the vulva is displaced.

10. A pessary which gives pain must at once be replaced by one which is painless.

11. A well fitting, properly chosen pessary should not only give no pain, but should be a direct source of comfort to the patient.

12. Always examine a patient on her feet after introducing a pessary, or when it is desired, at her return, to ascertain its efficiency in sustaining the uterus during walking and exertion.

13. Always tell the patient that she has a pessary in her vagina, or she may not return in spite of your directions, and the pessary may remain for years, to her ultimate great discomfort and danger.

14. Always tell the patient to return within a week after the first introduction, in order that the position and working of the pessary may be looked after, and that, if it does not suit, it can be removed and a better one inserted. Tell her that several trials and various instruments may be required before one is found which she can wear permanently. Also let her return for inspection once every four to eight weeks, as the case may require. Tell her that if she fails to do so the pessary may cause ulceration, for which treatment will be needed.

15. Tell the patient that she will need to wear the pessary for months, perhaps years, before a recovery can be expected.

16. Never introduce a pessary which a patient cannot remove herself.

17. Tell the patient to remove the pessary herself if it gives pain, and show her how to do it. When she has removed it, let her present herself at once for examination.

18. Tell the patient to use daily vaginal injections for cleansing purposes. If she notices profuse discharge, add astringents; if the discharge is sanious or purulent, let her come at once, as the pessary has probably caused abrasion.

19. Tell her, on removing the pessary, to test the result; that the permanence of the benefit obtained therefrom cannot be determined for several days or weeks.

20. Always direct your patients to relieve all superincumbent pressure on the pessary by a proper support of their skirts, and, if the displacement be anterior, aid the internal supporter by an abdominal (supra-pubic) pad.

All pessaries may be introduced in the knee-chest position when it it is desirable or possible to replace the uterus only in that position.

A Sims speculum elevates the perineum, the air enters and expands the vagina, and the pessary (chiefly in retroversion and prolapsus) is introduced by touch and sight, and the patient laid over on her left side. For aggravated retroversion and for prolapsus of ovaries or uterus, this position offers many advantages over the left semiprone decubitus. Care must be taken to remember that the position of the patient is reversed, and that the pessary must be introduced accordingly.

Dr. R. Beverly Cole, California, spoke after Dr. Mundé. He said the Hodge pessary was the first one invented which showed any knowledge of the anatomy of the female organs. He said no two pessaries were exactly alike. Dr. Albert Smith, of Philadelphia, had constructed one which had the peculiarity of Simpson's (also Hodges') hard rubber instrument. He then exhibited his pessary with the important modification of a spring support for retroversion. The great advantage claimed by Dr. Cole for this modification over others for this class of cases (retroversion) is that there is no risk of inflammation from the uterus having to impinge during the various movements of the person, upon a rigid, unyielding point. Dr. Cole thought that the numerous pessaries of Thomas were valueless for anteversion. In his hands the anteversion cup pessary of Thomas had failed, but Dr. Mundé, of New York, had used them successfully. Dr. Cole's hard-rubber pessary with spring modification has been most serviceable in anteversion.

Dr. Cole said he was not aware that a condition of the uterus, which he characterized as *atony*, had ever been described before. The organ becomes flabby and soft, settles down on the posterior wall of the vagina, and makes a double curve, the first forward, the second backward, and the os turned upward and forward on the posterior wall of the vagina. The Doctor illustrated this by a diagram on the blackboard. The upper curve often confuses the gynæcologist, and it is called sometimes a fibroid, sometimes the fundus of an anteflexed uterus. Great difficulty is often experienced in introducing the sound, and it may be often accomplished by turning the sound exactly in the opposite way, or its convexity backward. He had first commenced to treat them with an English bougie introduced every second day as an excitant to produce afflux of blood to the organ, and thus restore tonicity. Afterwards he got from Dr. James Simpson, of Edinburgh, the idea of the galvanic intra-uterine stem pessary, made above of zinc and below of copper. The intra-uterine stem galvanic pessary of Dr. Cole differs from that of Dr. Simpson in this important particular; the current in Dr. Simpson's is limited to the point of union of the two metals at the center of the staff, whilst Dr. Cole's, having the two metals arranged as plates running longitudinally, insulated by an interposing layer of hard rubber, distributes the current the *whole length of the stem.* This pessary is, according to Dr. Cole, in *super*-involution (atrophy) of the uterus, invaluable. By means of this instrument he has built up the uterus from its atrophied condition to one of health, brought back the menstrual function which had been absent for five years, and had the satisfaction of seeing pregnancy supervene.

Dr. Cole next exhibited an instrument of his invention which he called a gas cautery, for the cauterization of the mucous membrane of the cervical canal in cases of fungus growths thereon and other conditions. The Doctor advocates the use of *red* instead of *white* heat in these operations, as the *red* is less apt to cause troublesome hæmorrhage. He can produce a red heat in thirty seconds with this instrument.

He next showed Simpson's method of making pessaries to suit individual cases by moulding gutta-percha, previously softened in warm water, in the hands.

Dr. Chadwick next made the discussion general, and called upon Dr. Albert H. Smith, of Philadelphia, who said that, in his opinion, pessaries were too much in use, as they were often hurtful if not used judiciously. He said his pessary was only intended for retroversion and prolapsus. Anteversion, anteflexion and retroversion he had frequently relieved by means of the intra-uterine stem pessary, but he did not believe in the galvanic action. He thought it simply mechanical.

Dr. H. P. C. Wilson, of Baltimore, Maryland, agreed with Dr. Mundé, of New York, as regards the importance of ascertaining whether the uterus can be lifted up before introducing any pessary. He said he was often compelled to use a dozen or more before he could get one to fit with comfort. He remarked that he had once succeeded in relieving a case of anteflexion by introducing Hodges' horseshoe pessary. Dr. W. did not approve of stem pessaries. He said he had the best results in anteflexion from splitting the cervix backwards and the os internum backwards and forwards.

Dr. Maughs, of St. Louis, discussed the ligamentous supports of the uterus, and dilated upon the importance of the vaginal walls and perineum as supports. He said that, in his opinion, stem pessaries had killed hecatombs of women. He said anterior displacements were the rule in virgins, posterior in multipara.

Dr. Quimby, of Jersey City, said he had never derived any satisfaction from the use of stem pessaries, and said that he more and more inclined to avoid the use of all pessaries as he grows older.

Dr. Mundé was requested by the Chair (Dr. Chadwick, of Boston) to close the discussion, and rose to make some explanations, and was followed by Dr. R. Beverly Cole in a few closing remarks.

Adjourned till 3 P. M., May 4.

SECOND DAY.

SECTION ON OBSTETRICS AND DISEASES OF WOMEN.—Chairman, Dr. Jas. R. Chadwick, of Boston, meets at Wilkinson's Hall, at 3 o'clock P.M.; Secretary, Dr. Joseph Tabor Johnson, D.C.

Dr. F. C. Larrimore, of Ohio, opened the discussion on the Investing Capsule of Uterine Fibroids.

Dr. Dunster, of Ann Arbor, Mich., followed. He stated that there was, according to his experience, an investing capsule proper.

Dr. Dunlap, of Ohio, cited a case.

The Chair (Dr. Chadwick, of Boston) called on Dr. Burn, of Brooklyn, N. Y. Dr. Burn stated that he had examined a large number of uterine fibroids, both of the pediculated and sessile varieties, and utterly failed to find anything which he thought he could call a capsule proper. He said he was free to admit that he *always* found a more or less dense fibrous investment, varying with size and location, but considered this only the displacement or condensation of cellular tissue by the morbid growth or the result of hypertrophy.

Dr. Albert H. Smith, of Philadelphia, agreed with Dr. Burn, and spoke briefly in support of his position.

Dr. Erich, of Baltimore, considered it a matter of no consequence whether the investing membrane be called a capsule or not. There is always more or less of it.

Dr. Albert H. Smith, of Philadelphia, called on the Chair (Dr. Chadwick, of Boston) to give his experience. Dr. Chadwick declined.

Dr. Maughs, of St. Louis, cited a case operated on by Dr. J. Marion Sims, of New York. He said that when the tumor was removed, he was sure that all of the morbid growth had not been taken out, as a part of the mass showed an absence of the usual investing membrane. He said that a part of the uterine tissue adhering to the mass might have caused the same appearance. [Reporter could not hear whether he stated that the tumor returned or not.]

Dr. E. S. Dunster, of Ann Arbor, Mich., remarked that the discussion was only a war of words as long as no one defined precisely what an investing capsule is.

Dr. H. P. C. Wilson, of Baltimore, was requested by the Chair to exhibit the uterine dilators of his own construction. Dr. Wilson then rose and exhibited his instruments with a number of others of other makers. In the course of his explanatory remarks, he said that he considered the finger-nail the best curette. The dilators often enable the gynæcologist to dispense with tents, the use of which is usually avoided when it was practicable. He then cited the case of a virgin with intra-uterine fibroid, in whom he used a tent to begin the dilatation and followed it with the dilator; then after sufficient dilatation he removed the tumor with the curette of Thomas, with complete success.

Dr. Wilson thinks it very important to remove the placenta after abortion, or even normal labor, by instrumental means, if it does not come away spontaneously very soon. He advises the use of anæsthetics when this is to be done. He thought dilatation useful in atonic conditions of the uterus as a means of stimulating the organ.

Dr. R. Beverly Cole, of San Francisco, differed in toto, he said, with the last gentleman (Dr. Wilson.) He fears active inflammation from injury done in forcible dilatation. He dwelt on the fact that often serious trouble arises from very trivial injuries to this organ. He has known forcible dilatation to produce the severest forms of pelvic cellulitis and endometritis. The doctor does not think tents produce septicæmia if used discreetly.

Dr. Mundé, of New York, took issue with Dr. Cole, and supported Dr. Wilson's arguments in favor of his (Dr. Wilson's) instruments. He had often used them without bad results.

Professor King, of Washington, D. C., was then called on by Dr. Cole. Professor King thought that instrumental appliances were dangerous, and that the tendency was growing to resort too frequently to them. He advocated strongly giving nature a fair opportunity, and if it proved inadequate, he thought that the safer and milder methods should be tried first. Then, in case of failure, the physician, he said, could resort to the use of instruments. He said that often a purgative would cause an adherent placenta to be expelled if the patient were put in the upright position on a vessel to evacuate the rectum. Emetics often have the same effect by causing the muscular contractions incident to vomiting. A pinch of snuff, causing sneezing he said, would even effect the expulsion in some cases, if the nose were held during the act. He thought Hippocrates, with all the lights of the nineteenth century before him, would use, if he were still alive, some of his primeval methods in these cases before resorting to the brilliant and powerful array of instruments now in our armamentarium.

Dr. Erich, of Baltimore, took issue with Prof. King, but thought that he was almost too much afraid of instruments. He thought: "*In medias res tutissimus ibis.*"

Dr. Warner, of Massachusetts, agreed with Dr. Erich.

Dr. Robert Battey, of Georgia, agreed with Dr. Warner. He was of opinion that the tendency now-a-days was to resort to instrumental methods for too trivial causes and far too frequently. Dr. Battey said the "*vis medicatrix naturæ*," was often sufficient when it was not thought to be. He said Dr. Wilson's instruments were well enough, but it depended entirely upon the hand and the head that guided.

Dr. Albert A. Smith, of Philadelphia, does not approve of Dr. Wilson's dilators. He much prefers the tents. He says the instruments make small lesions about the cervical canal, hence the dangers of septicæmia, in careless hands. The tents, he thinks, are safer if not withdrawn before the end of forty-eight hours instead of twenty-four, as is usually done now. The tents ought to be *cylindrical* (not conical), and should be rubbed over with moist soap and dipped in a solution of salicylic acid before being introduced.

Dr. Maughs, of St. Louis, agreed with Dr. Smith. Thinks Dr. Wilson's instruments too dangerous. He says the finger can often be introduced into the uterus by placing the point at the os and assisting with the other hand externally on the abdomen. He advocated the use of tents very strongly.

Dr. H. O. Marcy, of Boston, defends tents used antiseptically.

Dr. H. P. C. Wilson replied to the arguments used against his instruments, that the objections urged by the gentlemen were rather against the injudicious use of than against his instruments.

Dr. H. O. Marcy, of Boston, at the request of the Chair, exhibited his double drainage and injection tubes. He showed in a very striking manner how they could be used as a means of applying warm water through the rectum in cases of pelvic cellulitis, or as a stomach-pump with Davidson's syringe. He also cited a case of cystitis, which was cured by keeping up a continuous stream of warm water through the bladder for twenty-four hours.

### THIRD DAY.

SECTION ON OBSTETRICS AND DISEASES OF WOMEN.

The Chair announced the following committees:

Committee for Selection of Subject of Essay—Prof. E. S. Dunster, Michigan; Prof. G. M. B. Maughs, St. Louis; Dr. H. M. Field, Boston.

Committee of Award—Dr. Robert Battey, Georgia; Dr. Albert H. Smith, Philadelphia, Pa.; Dr. P. F. Mundé, New York city.

Committee to which Papers for Publication are referred—Dr. Joseph Tabor Johnson, Washington, D. C.; Dr. John Byrne, Brooklyn, N. Y.; Dr. H. P. C. Wilson, Baltimore, Md.

*Can we Make a Positive Diagnosis of Pregnancy Previous to the Occurrence of the Audible Sounds of the Fœtal Heart and the Detection of the Fœtal Movements?* was the subject of an article by Dr. Joseph Tabor Johnson, of Washington, D. C. It is claimed that in the softened condition of the cervix uteri and the pinkish color and increased temperature of the vagina, we have quite positive diagnostic evidence of pregnancy. It is admitted that the only positive and indisputable signs are determined by auscultation, ballottement and fœtal movements; but these signs are not usually present in the first half of pregnancy. The presence of kiesteine in the urine, milk in the breast, the odor of vernix caseosa upon the finger as it is withdrawn from the vagina after a digital examination, the smooth condition of the anterior wall of the vagina and the anterior cul de sac, associated with a pinkish-purple color of the vaginal mucous membrane, the placental souffle, the existence of *gravidene* in the urine, the presence of certain caseous elements, resembling milk, in the urine, were all passed in review as diagnostic signs of pregnancy; but no definitely stated conclusions were arrived at.

Dr. R. Beverly Cole, of San Francisco, California, did not hear the whole of Dr. Johnson's paper, but thought from what he did hear, it was rather an interrogation than a treatise. He then stated that there were three physical signs of pregnancy which he relied chiefly upon, viz: 1st, placental souffle; 2d, pulsation of the cord, and 3d, the sounds of the fœtal heart. He regarded the last as the best and most reliable of them all. The pulsations vary from 110 to 140—double that of the mother. He described the sound as resembling the ticking of a watch under a pillow. Dr. Cole thought no signs positive enough (generally) to justify one in giving *decided* opinions when consulted on this point. He then cited a case, that of a girl who came to him and wished to ascertain whether or not she was pregnant. She appeared to be about six months advanced. Placental souffle was present, but no pulsation. The navel was protruding—admitted that she had intercourse with a man about six months before, and had not menstruated since. The Doctor then made an appointment for another examination, but she went to another physician, who examined her six times, called in some three or four others in consultation, who advised the introduction of a sound. This was done, and when it was withdrawn the discharge proved that it had penetrated into an abscess which Dr. Cole thought was undoubtedly due to pelvic cellulitis.

Dr. Albert H. Smith, of Philadelphia, thought placental souffle the most unreliable of the signs mentioned by Dr. Cole. He was anxious, he said, to hear Dr. Joseph Tabor Johnson's results from the thermometric observations on the cervix uteri. He thought Dr. Johnson's suggestions in regard to the application of the telephonic principle to the uterine sound, and the use of the electric light for illuminating the vagina, very good—quite brilliant, he would say. Liked the bi-manual method best of all.

Dr. Paul F. Mundé, of New York City, agreed with Dr. Smith. His favorite method was the bi-manual. He thought Dr. Smith had touched the key-note in making this statement. He thought that this method, taken with the other signs usually associated, would enable one to make out a case better than any other methods he knew of. He cited a case in support of his position.

Dr. Alex. Dunlap, of Ohio, agreed with Drs. A. H. Smith and Joseph Tabor Johnson. His method was the same, the bi-manual. The presence of fibroids may sometimes mislead as they enlarge the womb, but they are generally hard when small. Sometimes soft and dropsical when large, and rarely symmetrical. These points he thought are well to notice. Sanious discharge from the os is strong evidence of intra-uterine fibroid.

Dr. James M. Scott, of St. Louis, wanted to know if it was not a very difficult thing to use the bi-manual method on a fleshy patient, which was answered in the affirmative.

Prof. G. M. B. Maughs, of St. Louis, wanted to know if there were any signs by which we tell that a woman was *not* pregnant. (Dr. Joseph Tabor Johnson, of Washington, D. C., suggested, "Are there any signs by which we tell that a woman *is* pregnant?") Dr. Maughs adhered to the original form of his query. He thought that we have an almost certain method, viz., the bi-manual. He said that the measurement of the uterus laid down for the various weeks of pregnancy would enable us to determine after examining.

Prof. A. F. Erich, of Baltimore, disagreed with Prof. G. M. B. Maughs, of St. Louis, on the ground that the standards of measurement are too variable. He thought the thermometric indications in the os uteri would prove of great value as an aid in diagnosis.

Dr. Robt. Battey, of Georgia, arose and stated that he was surprised that no gentleman had mentioned any of the following aphorisms, in doubtful cases :

1st. Always consider a married woman pregnant if living with her husband until *proved* otherwise.

2d. Always consider an unmarried woman innocent till *proved* guilty.

3d. Always believe that a woman married, of the highest character, living with a husband of equally high character, both solemnly assuring the medical man that no intercourse has taken place for two years, as she has been bedridden for that length of time, may bring forth a dead fœtus.

4th. Always believe a young unmarried woman, with abdominal tumor, of high social position and unimpeachable virtue, if she has been watched over by a platonic and abstemious young cousin of the male persuasion, while the mother went out, to be pregnant.

Dr. Battey sat down amidst great applause and laughter.

Dr. James R. Chadwick (chairman), of Boston, drew a diagram, on the board, of the pelvic organs, and illustrated his methods of bi-manual examination.

Dr. Albert H. Smith, Philadelphia, drew a representation on the board of a thermometer made with an angle and guards for taking the temperature of the cervix.

Dr. J. R. Chadwick stated that he had noticed blueness of the vagina in many cases of pregnancy at various stages.

Dr. Alex. Dunlap, Ohio, is very careful about introducing the sound. He makes his digital examinations whilst the patient is standing.

Dr. —— Jennings, of ———, wanted to know whether the uterine normal temperature was not higher than the rectal or sublingual.

Dr. A. H. Smith replied that it was not in pathological (except inflammation) or the healthy, unimpregnated states.

Dr. Johnson asked Dr. Dunlap to give his experience as to the blue color of the vagina in cases of fibroids.

Dr. Dunlap said that as he rarely used the speculum, he had none.

Dr. Johnson closed by making a few remarks on the subject, which the reporter failed to get.

Dr. Paul F. Mundé, New York city, exhibited a curette for the removal of adherent placentæ after abortion, designed by himself. Dr. Mundé also exhibited a speculum with a flange for holding up the buttock, which he had had made; also two smaller curettes. He said he thought it best to remove the placenta reasonably soon if it did not come away in cases of abortion.

Here followed a long discussion on this point, which was participated in by Drs. Maughs, Dunlap, Marcy, Smith, Johnson, Erich, Chadwick and G. McDonald, who said he was a "country doctor," and always removed them at once by means of instruments if he could not do so with the finger, as he could not see his patients often or quickly enough in dangerous cases. The other gentlemen agreed pretty nearly with Dr. Mundé.

Dr. Mundé closed.

Adjourned.

